# Performance of the Ionospheric Kappa‐Correction of Radio Occultation Profiles Under Diverse Ionization and Solar Activity Conditions

**DOI:** 10.1029/2020EA001581

**Published:** 2021-06-14

**Authors:** J. Danzer, S. J. Haas, M. Schwaerz, G. Kirchengast

**Affiliations:** ^1^ Wegener Center for Climate and Global Change (WEGC) University of Graz Graz Austria; ^2^ Institute of Physics NAWI Graz University of Graz Graz Austria

**Keywords:** kappa‐correction, radio occulation, residual ionospheric errors, stratospheric data quality

## Abstract

The kappa‐correction is an easy‐to‐use method to correct for residual ionospheric errors in radio occultation (RO) data. It is a simple empirical model term that only depends on readily available data. While its basic utility was well proven in previous studies, including a recent predecessor study on RO climatologies under solar cycle variations, its performance for individual RO profile correction under diverse and extreme ionization conditions is unclear so far. Here we tackle this gap and focus on investigating (extremely) low and high solar activity and ionization conditions of individual RO events, including inspection of ionospheric symmetry between inbound and outbound raypaths. Using a global multi‐year ensemble of MetOp‐A and GRACE‐A RO events over 2008–2015 as basis, we applied a sampling approach leading to six characteristic condition cases. These cases also relate to day and night time variations and geographic variations from the equatorial to the high latitude region. We inspected the kappa‐correction and its performance relative to the standard bending angle correction for RO‐retrieved stratospheric profiles and found mean deviations in temperature of near −0.3 K in the upper stratosphere (40–45 km) for high ionization conditions, with extreme deviations exceeding −2 K for strong inbound/outbound asymmetry. The kappa‐correction term itself reaches a mean value near 0.05 μrad under these high conditions. Low solar activity and ionization conditions lead to a mean correction smaller than 0.005 μrad and mean temperature deviations smaller than 0.02 K. An intercomparison to other quality datasets, predominantly showed a decrease in mean temperature difference when applying the kappa‐correction.

## Key Points

- The kappa‐correction of radio occultation profiles is explored for low and high ionization, solar activity, and asymmetry
- The mean correction is most relevant under high ionization and symmetry, reaching near 0.05 μrad around 50 km
- Intercomparison with other datasets shows it predominantly decreases retrieved stratospheric temperature errors

## 1 Introduction

Over the past years the global navigation satellite system (GNSS) radio occultation (RO) technique (Hajj et al., 2002; Kursinski et al., 1997) has become of increasing importance for climate and meteorological applications (Anthes, 2011; Cardinali, 2009; Cucurull, 2010; Healy & Thépaut, 2006; A. Steiner et al., 2001; A. Steiner et al., 2011). It provides a continuous record of near‐vertical geophysical data profiles, since the launch of the CHAllenging Minisatellite Payload (CHAMP) mission in the year 2001 (Ao et al., 2003; Foelsche et al., 2003; Wickert et al., 2001). RO data show highest accuracy in the upper troposphere and lower stratosphere between about 5–35 km (Foelsche et al., 2011; Kursinski et al., 1997; Zeng et al., 2019). Enabling improved accuracy of RO data also at higher altitudes, where global and long‐term data are relatively scarce, results in improved value also as data source for numerical weather prediction (NWP), climate monitoring, and new stratospheric climate analyses such as, for example, deriving stratospheric wind fields (Healy et al., 2020; Scherllin‐Pirscher et al., 2014, 2017).

Toward increasing altitudes, RO bending angles have a decreasing signal‐to‐noise ratio, due to an increasing impact of measurement noise and ionospheric refraction in the data. In the retrieval processing chain, the related errors propagate downward from RO bending angle (*α*) to refractivity (*N*), pressure (*p*), and temperature (*T*) (Gobiet et al., 2007; Ho et al., 2012; Rieder & Kirchengast, 2001; Schwarz et al., 2017, 2018; A. Steiner et al., 2013; A.K. Steiner & Kirchengast, 2005). In this specific work, data quality is increased in the middle and upper stratosphere by applying a second‐order ionospheric correction on the bending angle profiles (Healy & Culverwell, 2015), the so‐called kappa‐correction. The focus lies on understanding the second‐order impact on the dry atmospheric RO parameters (*α*, *N*, *p*, *T*), and investigating its variation under diverse and extreme solar activity and ionization conditions, since this is an important gap left by previous studies (introduced further below).

The primary measured quantity in the RO technique is the excess phase path profiles at the two Global Positioning Satellites L‐band carrier frequencies, *f*_1_ = 1575.42 MHz and *f*_2_ = 1227.60 MHz. From these excess phase profiles the bending angle profiles $\alpha_{L_{1}}$ and $\alpha_{L_{2}}$ can be derived, which are then combined using a dual‐frequency linear combination of the RO bending angles, in order to correct for the influence of the ionosphere to first‐order (Ladreiter & Kirchengast, 1996; Vorob'ev & Krasil'nikova, 1994). The remaining higher‐order residual ionospheric errors (RIE) in the RO data are of increasing importance with increasing altitude (above about 35 km); furthermore they vary (mainly) with the diurnal and solar cycle (Danzer et al., 2013; Liu et al., 2013, 2015, 2018; Mannucci et al., 2011; Syndergaard, 2000). Earlier approaches for higher‐order ionospheric corrections exist (Hoque & Jakowski, 2008; Kedar et al., 2003; Syndergaard, 2002; Vergados & Pagiatakis, 2010, 2011), however, they are usually in need of additional background information, such as the total electron content (TEC).

More recently a second‐order ionospheric correction was introduced by Healy and Culverwell (2015), the so‐called kappa‐correction, which was at the same time also evaluated through simulation studies by Danzer et al. (2015). The kappa‐correction in its simple functional‐model form, introduced by Angling et al. (2018) as an advancement to Healy and Culverwell (2015), has the advantage of only needing the F_10.7_ index as auxiliary background information. Otherwise, it just depends on the retrieved $\alpha_{L_{1}}$ and $\alpha_{L_{2}}$ bending angle profiles, which are available from the RO processing, and the location and time of the RO profile data, capturing location‐ and time‐dependent solar variations.

In a recent predecessor study by Danzer et al. (2020), that used longer‐term real RO data, the correction was tested for its influences on RO‐derived climatologies as well as validated against reanalysis datasets. Analyzing 10° zonal‐mean climatologies from the solar minimum year 2008 to the solar maximum year 2014, the study found that the kappa‐correction generally warms the RO temperature climatology data. Furthermore, it showed a sensitivity of the kappa‐correction of less than 0.2 K for low and more than 0.6 K for high solar activity conditions, in a middle stratosphere layer (30–35 km), with the largest correction over the tropics (20°S‐20°N). The validation analysis showed that it is challenging to validate small improvements of RO data; datasets used were from the European Center for Medium‐Range Weather Forecasts (ECMWF) reanalyzes ERA‐Interim (Dee et al., 2011) and ERA5 (Hersbach et al., 2018, 2020). It was found difficult to discern small improvements in the RO data, since the validation datasets also feature small biases that are of similar magnitude as the ionospheric RIE correction term. The problem of validating improvements with other datasets will likely continue for other proposed changes to GNSS RO processing in the future.

Another recent study by Liu et al. (2020) provided a first assessment of a further advanced higher‐order RIE correction, the so‐called bi‐local correction (Syndergarrd & Kirchengast, 2019), which on top of the kappa‐correction's scope accounts also for the geomagnetic higher‐order term, the finite RO receiver orbit altitude, and ionospheric inbound/outbound asymmetry. It requires significantly more auxiliary background information, such as the TEC for the inbound and outbound regions of each RO event. The initial intercomparison of the bi‐local correction with the kappa‐correction under different ionization conditions by Liu et al. (2020) showed that the latter is, in spite of its simplicity, generally very comparable and consistent with the more advanced correction, with limits reached for smaller‐scale averages and under individual‐event conditions that are not captured by its more simplified formulation. Hence, it is valuable to further explore the kappa‐correction performance especially also for diverse and extreme solar and ionization conditions.

In this study we focus on a kappa‐correction performance analysis based on ensembles of individual RO events, applying a targeted subset‐sampling approach to a large global multi‐year ensemble of RO data. The concept is to subsample all profiles that occur beyond particular thresholds of solar activity (measured in daily F_10.7_ values), ionization level (measured in vertical total electron content vTEC), and degree of inbound/outbound asymmetry (measured by an asymmetry factor *f*_*IA*_ introduced in Section 2). More specifically, we use all MetOp‐A and GRACE‐A RO events from the years 2008–2015 as basis, from which we subsample those which occurred during specific high and low F_10.7_, vTEC, and *f*_*IA*_ conditions.

This approach has the advantage to extract robust subsets of RO profile data for ensemble inspection and statistical analysis under very distinct ambient conditions of interest. Furthermore it intrinsically samples the diurnal (local time) cycle and the equatorial to midlatitude to polar regions in a characteristic and insightful way (as seen in Section 2 on methods and datasets).

Hence the approach enables to inspect the performance of the kappa‐correction explicitly under low and high solar activity, ionization, and asymmetry conditions and implicitly under diurnal and solar cycle variations as well as geographical variations, capturing the most salient temporal and spatial variations of the ionosphere. Closer analysis of these specific variations of RIEs was recommended also in International Radio Occultation Working Group (IROWG) climate subgroup recommendations (https://irowg.org/irowg7_minutes_summary/, last access 28 October 2020). This study therefore contributes also to meet this recommendation.

The paper is structured as follows. After introducing the method and datasets in Section 2 we investigate and discuss the kappa‐correction's performance related to RO bending angle, refractivity, pressure, and temperature profiles (Section 3.1), complemented also by detailed result summary tables in Appendix A. The kappa‐correction is afterward validated against other datasets from ERA5 and ERA‐Interim reanalyzes (Section 3.2). Conclusions are drawn in Section 4.

## 2 Method and Datasets

The impact of the ionosphere on the RO profiles is basically corrected in the Wegener Center (WEGC) RO processing that is employed here by applying the first‐order ionospheric bending angle correction given by Vorob'ev and Krasil'nikova (1994), also referred to as “standard correction” hereafter:

(1) $$\alpha_{c}\left(z_{a}\right)=\alpha_{L_{1}}\left(z_{a}\right)+\frac{f_{2}^{2}}{f_{1}^{2}-f_{2}^{2}}\left[\alpha_{L_{1}}\left(z_{a}\right)-\alpha_{L_{2}}\left(z_{a}\right)\right].$$

In this equation *α*_*c*_ is the estimate of the neutral atmosphere bending angle in [rad], and $\alpha_{L_{1}}$ and $\alpha_{L_{2}}$ are the *L*_1_ and *L*_2_ bending angles ([rad]) related to the frequencies *f*_1_ and *f*_2_ ([Hz]), given at impact altitude *z*_*a*_ ([m]). Healy and Culverwell (2015) proposed a modification to the standard ionospheric correction, with an additional (positive) term to compensate for the higher‐order ionospheric error:

(2) $$\alpha_{c}\left(z_{a},t\right)=\alpha_{L_{1}}\left(z_{a}\right)+\frac{f_{2}^{2}}{f_{1}^{2}-f_{2}^{2}}\left[\alpha_{L_{1}}\left(z_{a}\right)-\alpha_{L_{2}}\left(z_{a}\right)\right]+\left|\kappa ,\left(z_{a},t\right)\right|\cdot {\left[\alpha_{L_{1}}\left(z_{a}\right)-\alpha_{L_{2}}\left(z_{a}\right)\right]}^{2}.$$

The latter term is the kappa‐correction term, depending only on a slowly varying kappa factor *κ*(*z*_*a*_, *t*), and the $\alpha_{L_{1}}$ and $\alpha_{L_{2}}$ bending angle profile difference squared, which approximately models the dominant residual ionospheric variation from profile to profile. The kappa factor *κ*(*z*_*a*_, *t*) ([rad^−1^]) is expressed by Angling et al. (2018):

(3) $$\kappa \left(z_{a},t\right)=a+b\cdot F_{10.7}\left(t\right)+c\cdot \chi \left(t\right)+e\cdot z_{a}.$$

The kappa factor depends on the F_10.7_ index, given in solar flux units [sfu], and on the solar zenith angle *χ*(*t*), given in [rad], which contains the information of local time, season, and location of a profile. Furthermore, the kappa factor exhibits a slow altitudinal variation represented by the dependence on impact altitude *z*_*a*_. *a*, *b*, *c*, *e* are regression coefficients found by fitting the model to large datasets (Angling et al., 2018).

### 2.1 Sampling Approach and Characteristic Condition Cases

In order to analyze the impact of the natural ionospheric variations on the residual ionospheric error (RIE), we apply a sampling approach on the individual RO profiles. The goal is to assess diurnal cycle, solar cycle, and geographical variations, as well as ionospheric raypath inbound and outbound asymmetry effects, on the kappa‐correction.

**Figure 1:**
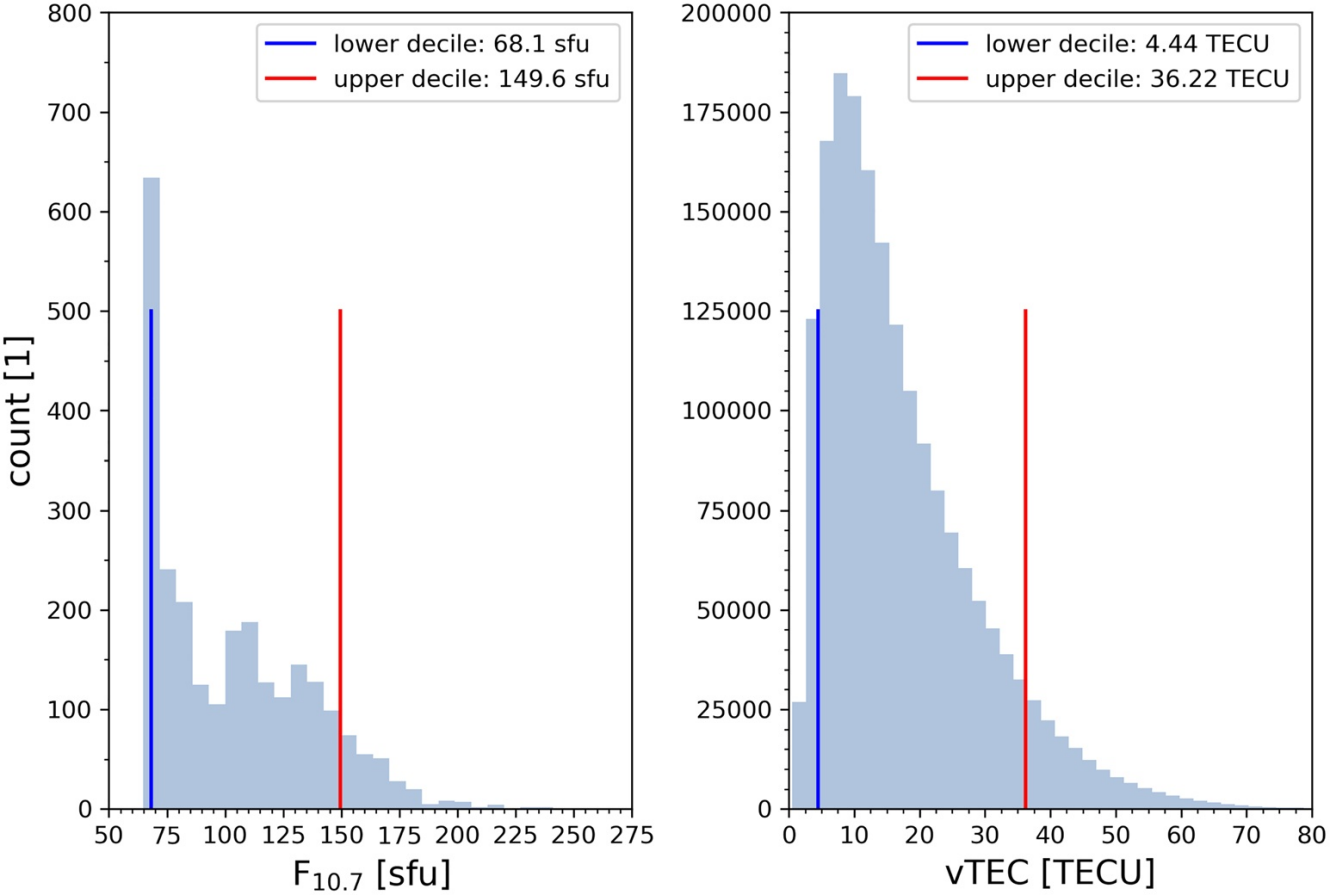
Histogram of the daily F_10.7_ index values (left) and RO‐event‐collocated vTEC values (right), supporting the definition of the condition classes (Table 2), using the data from the solar minimum year 2008 up to the solar maximum year 2014. The blue and red lines mark the lower (blue) and upper (red) decile (i.e., 10*%* and 90*%* percentiles).

As a first diagnostic plot we inspect daily F_10.7_ values (unit [sfu], 1sfu = 10^−22^Wm^−2^ Hz^−1^) from the solar minimum year 2008 up to the solar maximum year 2014 (Figure 1, left panel). We intend to find the lower and upper 10*%* (decile) of days within the minimum to the maximum of the solar cycle, in order to distinguish low solar activity from high solar activity (In the later analysis plots when investigating RO data, we also include the year 2015 in the data range, in order to achieve a high profile statistics). We round the results for the two deciles to F_10.7_ = 70 sfu, and classify all days up to this value as low solar activity days, while days with F_10.7_ = 150 sfu or higher are classified as high solar activity days. We apply the same diagnostics to the electron density, for classifying into low and high ionization conditions, by using the information of vTEC (unit [TECU], 1 TECU = 10^16^ electrons per m^2^) (Figure 1, right panel). From these results we choose for the subset sampling event‐collocated vTEC values of vTEC = 5 TECU and below for classifying RO events into low ionization conditions and vTEC values of vTEC = 35 TECU and higher for classifying into high ionization conditions, respectively.

**Figure 2:**
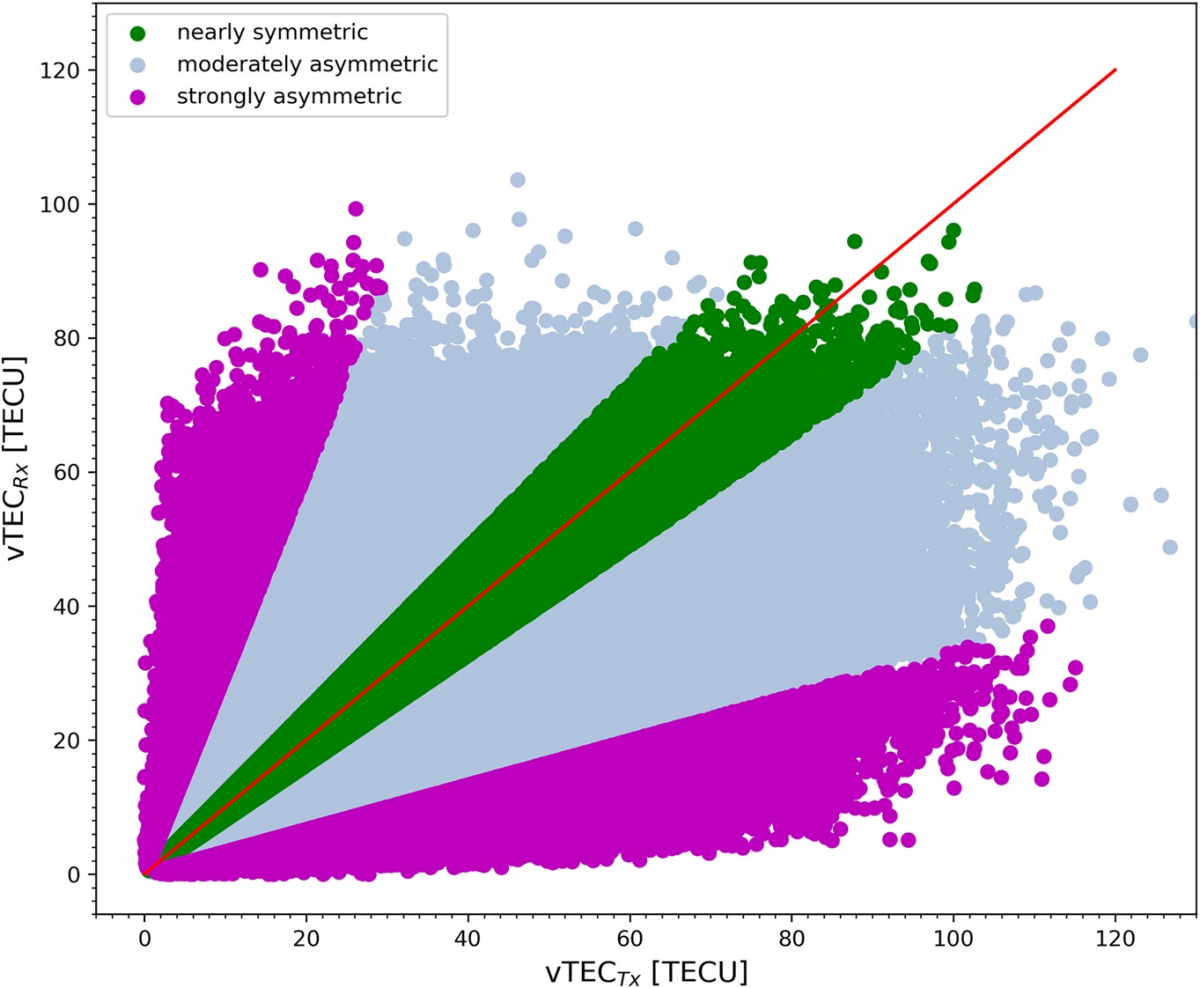
Scatter plot of the electron density showing inbound (vTEC_*Tx*_) versus outbound (vTEC_*Rx*_) conditions for the MetOp‐A data over 2008–2014. The colors classify the ionospheric conditions, according to Equation 4, into nearly symmetric (green), moderately asymmetric (blue), and strongly asymmetric (purple) RO event sub‐ensembles.

Since the ionospheric correction does not account for large‐scale horizontal electron density gradients in the ionosphere along the GNSS signal's inbound and outbound raypaths, we also distinguish inbound/outbound‐symmetric and ‐asymmetric conditions, in order to inspect the sensitivity of the kappa‐correction also in this respect. Figure 2 illustrates the vTEC between inbound (from GNSS transmitter) and outbound (toward LEO receiver), respectively, defined as vTEC_*Tx*_ (inbound, *x*‐axis) and vTEC_*Rx*_ (outbound, *y*‐axis), respectively (for discussion of detailed RO event examples and causes of such asymmetries see, e.g., Liu et al. (2013, 2018)). For enabling classification into nearly‐symmetric and strongly asymmetric cases we used the following formal definition of an ionospheric asymmetry factor, in line with Liu et al. (2020), which here just serves this particular classification task:

(4) $$f_{IA}\left[\%\right]=100\cdot \frac{{vTEC}_{Tx}-{vTEC}_{mean}}{{vTEC}_{mean}},$$

where vTEC_*mean*_ = 0.5 ⋅ (vTEC_*Tx*_ + vTEC_*Rx*_), and *f*_*IA*_ is the ionospheric asymmetry factor. A deviation of |*f*_*IA*_| ≤ 10% from the average inbound‐outbound vTEC is used to classify RO events into nearly symmetric conditions, while a deviation of |*f*_*IA*_| ≥ 50% comprises those under strongly asymmetric conditions.

**Figure 3:**
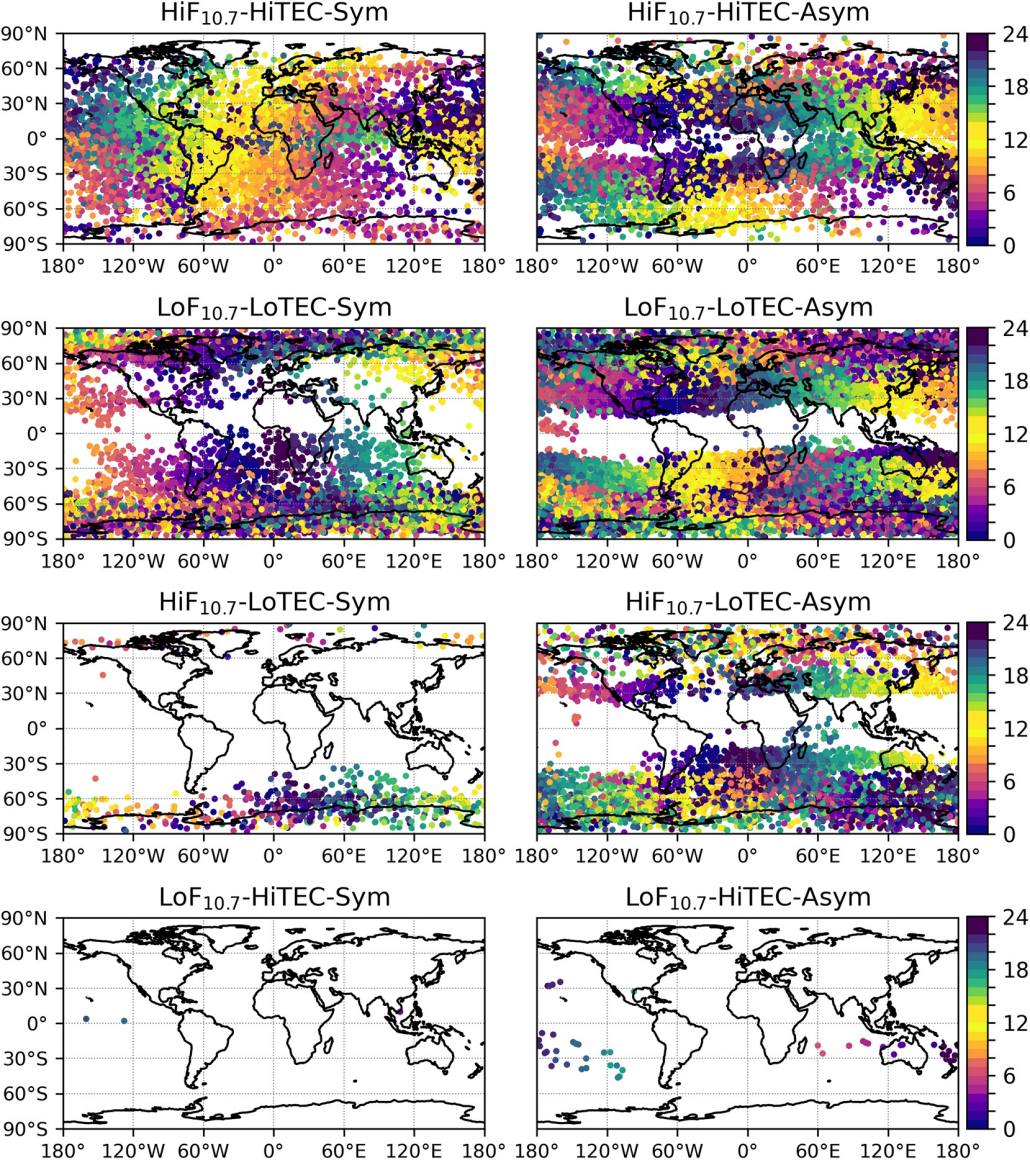
Geographic scatter plot map of the RO events of all eight condition cases (see Table 2 for the case names and conditions), analyzed for the MetOp‐A events from the years 2008 up to 2015. The RO events are marked as dots at their mean event location and the color indicates the local time at occurrence of the event (color bars on right‐hand side).

To summarize, we sample RO profiles according to the three condition parameters, F_10.7_ index, vTEC value, and *f*_*IA*_ factor, which is covered by the definition of conditions collected in Table 1. Though partly correlated with the vTEC, the F_10.7_ index provides additional information to the vTEC in our setup, since it enables improved discrimination between solar cycle and diurnal cycle effects. This leads to combinations of 8 different ensemble cases, summarized in Table 2. Since low F_10.7_ conditions do essentially not mix with the occurrence of high vTEC values (Figure 3, bottom row), we practically end up with six characteristic condition cases, distinguished by their ionization and solar activity level, with the advantage of a rather large ensemble of RO profiles for each case.

**Table 1:** Definition of Solar, Ionization, and Asymmetry Conditions

**Parameter**	**Weak condition**	**Strong condition**
F_10.7_ [sfu]	≤ 70 sfu, low solar activity	≥ 150 sfu, high solar activity
vTEC [TECU]	≤ 5 TECU, low ionization	≥ 35 TECU, high ionization
|*f*_*IA*_| [%]	≤ 10%, nearly symmetric	≥ 50%, strongly asymmetric

**Table 2:** Definition of Characteristic Condition Cases

**Case name**	**No. of events**	**Description**
HiF_10.7_‐HiTEC‐Sym	6156 (624)	high solar activity, high ionization, nearly symmetric
HiF_10.7_‐HiTEC‐Asym	8875 (1690)	high solar activity, high ionization, strongly asymmetric
LoF_10.7_‐LoTEC‐Sym	6413 (639)	low solar activity, low ionization, nearly symmetric
LoF_10.7_‐LoTEC‐Asym	23,908 (3756)	low solar activity, low ionization, strongly asymmetric
HiF_10.7_‐LoTEC‐Sym	515 (43)	high solar activity, low ionization, nearly symmetric
HiF_10.7_‐LoTEC‐Asym	4005 (539)	high solar activity, low ionization, strongly asymmetric
LoF_10.7_‐HiTEC‐Sym	3 (0)	low solar activity, high ionization, nearly symmetric
LoF_10.7_‐HiTEC‐Asym	46 (58)	low solar activity, high ionization, strongly asymmetric

*Note*: As Shown in Column 2, which gives the number of MetOp‐A events complemented by the number of GRACE‐A events in parentheses, the Cases LoF_10.7_‐HiTEC‐Sym/Asym exhibit a very small ensemble size (see also Figure 3). These cases are therefore dismissed in the following analysis. The total sample size of MetOp‐A (GRACE‐A) profiles is ∼1,458,100 (∼323,100) profiles from 2008 to 2015, so small fractions (<0.1*%* − 2*%*) of well‐defined extreme conditions are isolated here.

Figure 3 also illustrates the geographical mapping of all six characteristic condition cases. The HiF_10.7_‐HiTEC‐Sym/Asym cases (first row) occur primarily between about ± 60°N/S, while the LoF_10.7_‐LoTEC‐Sym/Asym cases (second row) do not occur over the equatorial region, but exist up to the northern and southern poles. The same is also true for the mixed cases of HiF_10.7_‐LoTEC‐Sym/Asym (third row).

Finally Figure 3 also illustrates, as a further information, the local time of each RO event by way of a gradual color bar. This indicates that we clearly observe a diurnal cycle mapping for the six characteristic condition cases. The HiF_10.7_‐HiTEC‐Sym/Asym cases strongly relate to daytime conditions, especially under ionospheric inbound/outbound symmetry, and are rare at polar latitudes. On the other hand, the LoF_10.7_‐LoTEC‐Sym/Asym cases show a majority of nighttime events with low vTEC values and are rare at equatorial latitudes. The “mixed” cases HiF_10.7_‐LoTEC‐Sym/Asym primarily occur during nighttime, when the ionospheric E‐layer vanishes and only the F‐layer remains, and cluster at middle to high latitudes.

### 2.2 Datasets

We used data from the Meteorological Operational Satellite (MetOp‐A) (Loiselet et al., 2000; Montenbruck et al., 2008; Von Engeln et al., 2009), which delivers data since the end of 2007. MetOp‐A covers the period of our investigated solar cycle (2008–2015) with a reliable profile statistics number of about 700 profiles per day. As a complementary RO dataset we used data from the Gravity Recovery and Climate Experiment (GRACE) (Beyerle et al., 2005; Wickert et al., 2005), using the GRACE‐A mission data in the same time range. For the processing we used the Wegener Center (WEGC) reference occultation processing system (rOPS) (Danzer et al., 2018; Innerkofler et al., 2020; Kirchengast et al., 2016, 2018; Liu et al., 2020; Schwarz et al, 2017, 2018) (V. Proschek et al., WEGC Graz, rOPS refractivity and dry‐air retrieval manuscript in preparation, pers. communications, 2021). MetOp‐A and GRACE‐A differ in their orbit altitudes, i.e., MetOp‐A orbits at an altitude of about ∼820 km, while GRACE‐A orbits at an altitude of about ∼470 km.

We applied the ionospheric kappa‐correction to all RO profiles, to use them for assessment against the RO profiles with standard correction, and used the sampling approach to classify the data subsets. For the daily F_10.7_ values we downloaded the data from Natural Resources Canada (https://www.spaceweather.gc.ca/solarflux/sx-en.php, last access: 18 February 2021). The vTEC maps were downloaded from the International Global Positioning System Service (IGS) center (https://kb.igs.org/hc/en-us/articles/115003935351, last access: October 28, 2020).

Using the F_10.7_ and vTEC datasets (the latter including inbound and outbound vTEC's, as needed for Equation 4), we sampled the RO profiles according into their respective category (i.e., low (Lo) or high (Hi) F_10.7_ and vTEC values, and Sym/Asym *f*_*IA*_ values). We analyzed the RO datasets with typically inspecting the difference between the profiles with the higher order kappa‐correction applied (radiolabeled as RO_*κ*_) against the profiles with just the first‐order standard correction applied (radiolabeled as RO).

For the intercomparison analysis with other quality datasets we used the European reanalyzes ERA5 and ERA‐Interim. In order to assess whether the kappa‐correction improves the RO profiles, the comparison datasets need to show a very high quality at stratospheric altitudes. We consider the chosen reanalysis datasets to fulfill this requirement in a best possible manner though still marginally. However, based on initial previous validation studies by Liu et al. (2019), including SABER infrared limb sounder data, and by Danzer et al. (2020), including MIPAS middle‐atmosphere infrared limb sounder data, we found these other observational satellite data are not sufficiently accurate.

Both the recent ERA5 reanalysis (Hersbach et al., 2018, 2020) and the predecessor reanalysis ERA‐Interim (Dee et al., 2011) involve a four‐dimensional variational data assimilation approach (4D‐Var), based on the integrated forecasting system IFS of the European Center for Medium‐Range Weather Forecasts (ECMWF). ERA5 used an improved horizontal resolution of about 30 km and 137 vertical levels from the surface up to 0.01 hPa (∼80 km), while ERA‐Interim used a resolution of about 80 km and 60 vertical levels up to 0.1 hPa (∼60 km). Both datasets fully cover the needed time period of 2008–2015.

Apart from the resolution and other model physics refinements, some differences in stratospheric temperatures from ERA5 compared to ERA‐Interim are induced by advanced background covariance matrices, a changed bias adjustment for radiosonde data, and assimilation of COSMIC GNSS‐RO bending angles from mid‐2006 onwards (ERA‐Interim from late‐2009 onwards) (Hersbach et al., 2020). Overall slightly colder stratospheric temperatures are found in ERA5, which leads to ERA‐Interim temperatures being in general globally about 1.5 K warmer than ERA5 near the stratopause (at 1 hPa level). Furthermore, there exists a larger warm bias near the stratopause.

## 3 Results

### 3.1 Kappa‐Correction Influence on Atmospheric RO Profiles

**Figure 4:**
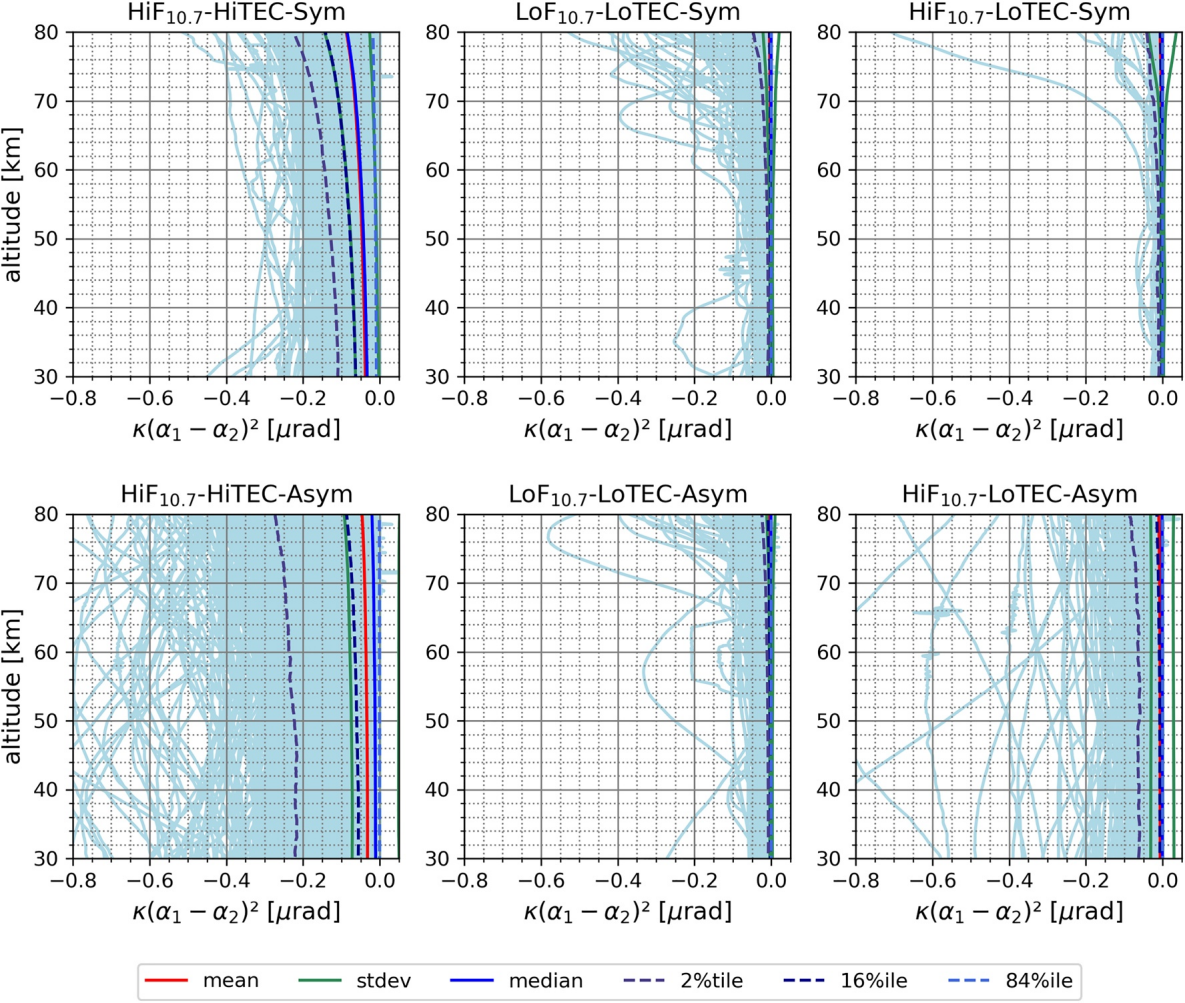
Size of the kappa‐correction term in RO bending angle profiles (MetOp‐A) over the upper stratosphere and mesosphere (30–80 km), comparing the six main characteristic condition cases of Table 2 (see panel titles for case identification; nearly‐symmetric cases top, strongly‐asymmetric cases bottom). For explanation of the six depicted statistics metrics, from mean to three selected percentile profiles, see the legend (bottom).

**Figure 5:**
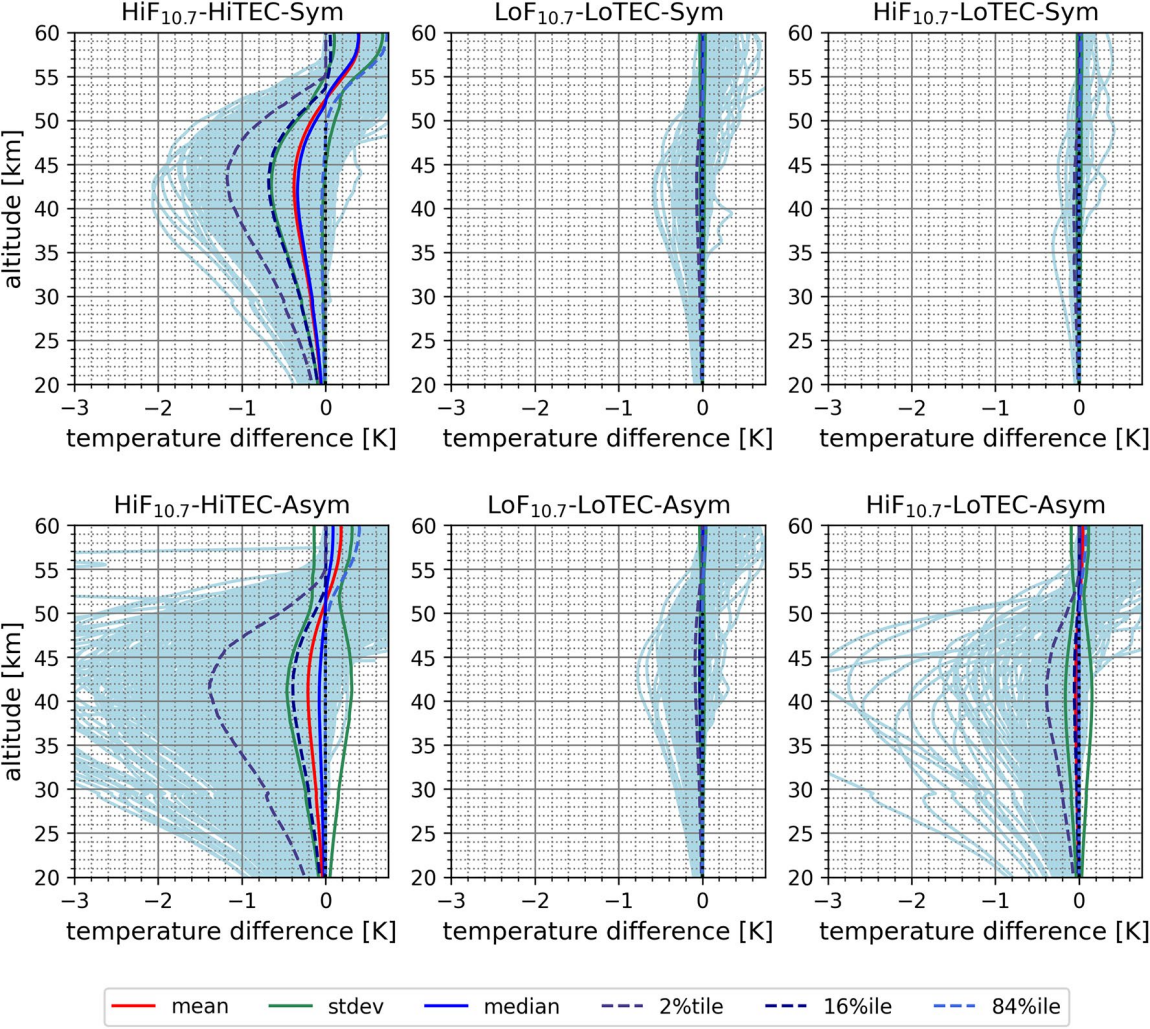
Kappa‐correction‐induced RO temperature profile deviations versus temperature profiles from standard bending angle correction (MetOp‐A) from lower stratosphere to mesosphere (20–60 km), comparing the six main characteristic condition cases of Table 2 (see panel titles for case identification; nearly‐symmetric cases top, strongly‐asymmetric cases bottom). For explanation of the six depicted statistics metrics, from mean to three selected percentile profiles, see the legend (bottom).

Here we present the overall kappa‐correction performance results for the six characteristic condition cases. Figure 4 shows the magnitude of the correction term itself for a bending angle profile‐to‐profile validation, while Figure 5 shows the impact for the temperature profile‐to‐profile validation, based on the WEGC retrieval processing (Subsection 2.2), illustrated for MetOp‐A RO data ensembles.

The size of the kappa‐correction term is clearly largest under high solar activity and high ionization (HiF_10.7_‐HiTEC) under symmetric and asymmetric conditions (Figure 4, left), with extreme individual‐profile deviations exceeding −0.2 μrad under nearly symmetric conditions and even −0.4 μrad under strongly asymmetric conditions. However, the mean value is found restricted to about −0.03 to −0.05 μrad over the stratosphere and, interestingly, markedly smaller under asymmetric conditions. For low solar activity and ionization conditions, the mean correction is smaller than −0.005 μrad. These mean results are in line with previous studies based on simulated data (Liu et al., 2015) and small observed data ensembles (Liu et al., 2020) and hence consolidate confidence in them, given the targeted large‐size ensembles used here. The specific behavior revealed under different levels of ionization asymmetry, contrasting on an individual RO profile basis the kappa‐correction term's mean‐size behavior, is an interesting new finding that points to the need of future more detailed investigation under specific regional‐scale conditions, as also recently suggested by Liu et al. (2020).

Using WEGC's rOPS refractivity and dry‐air retrieval chain, the retrieved stratospheric temperature profiles show deviations of near −0.3 K from including the kappa‐correction, for high ionization conditions in the upper stratosphere (40–45 km). In these cases, strong inbound/outbound asymmetry leads to salient individual‐profile deviations exceeding −2 K (−1 K under symmetry) (Figure 5). The correction‐induced temperature profile deviation increases with increasing altitude up to stratopause near heights (around 50 km), and decreases beyond into the mesosphere. This kind of propagating ​of the deviation is an effect of dry‐air retrieval processing (i.e., combined effect of hydrostatic integral and equation of state) as has been discussed by Schwarz et al. (2017) as part of introducing the rOPS uncertainty propagation from bending angle to dry‐air temperature profiles. For the low solar activity and mixed case the impact of the error is rather small at both levels. In the LoF_10.7_‐LoTEC‐Asym and LoF_10.7_‐LoTEC‐Sym cases, the mean temperature error in the upper stratosphere (40–45 km) is smaller than −0.001 K and hence negligible.

**Figure 6:**
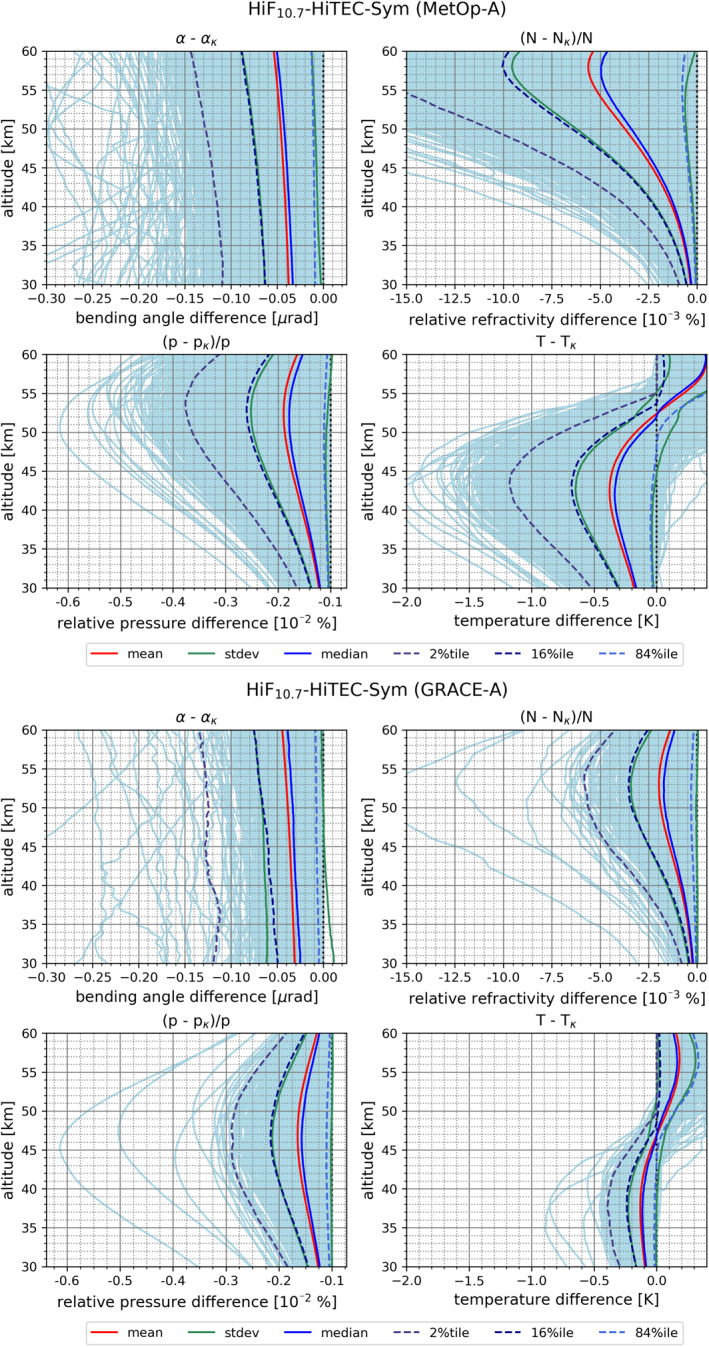
Kappa‐correction‐induced RO bending angle, refractivity, pressure, and temperature profile deviations versus standard‐correction, showing the case for high solar and ionization conditions and near‐symmetry (HiF_10.7_‐HiTEC‐Sym) for MetOp‐A (top part, upper four panels) and GRACE‐A (bottom part, lower four panels), respectively. For explanation of the six depicted statistics metrics, from mean to three selected percentile profiles, see the legend (bottom).

Figure 6 illustrates the kappa‐correction influence across the set of retrieved atmospheric profiles from bending angle to temperature, focusing on high solar and ionization conditions under near‐symmetry where the mean deviations are strongest. Additionally we show both the mission ensembles from MetOp‐A (upper part) and GRACE‐A (lower part). This highlights how the kappa‐correction‐induced deviations propagate through the retrieval processing chain from bending angle (*α*) via refractivity (*N*) and pressure (*p*) to temperature (*T*). The GRACE‐A ensemble exhibits a somewhat smaller diversity and spread of kappa‐correction deviations than MetOp‐A, which is likely related mostly to the overall smaller ensemble of profiles (Table 2), capturing less extreme events.

The mean correction‐term size at bending angle level is still very similar for both satellite missions (∼−0.03 to −0.05 μrad). However, at temperature level we find that GRACE‐A exhibits a smaller temperature deviation (e.g., GRACE‐A ∼−0.1 K), MetOp‐A (∼−0.2 K, at around 35 km). Furthermore, the altitude level of the sign switch of the temperature deviation from negative to positive is about 5 km lower for GRACE‐A (around 47 km, MetOp‐A around 52 km). Both these effects of “damping down” the GRACE‐A retrieved deviations compared to MetOp‐A are presumably mainly induced by the different weighting of observation and background information in the bending angle statistical optimization step of the retrieval process before the Abelian transformation to refractivity, where MetOp‐A receives highest weights of observed bending angles due to these data being assessed to have smallest observational errors (Angerer et al., 2017; Schwarz et al., 2017). This behavior is hence, as expected given that these different RO mission data properties apply in general, also found for the other characteristic cases including asymmetric conditions (not shown).

**Figure 7:**
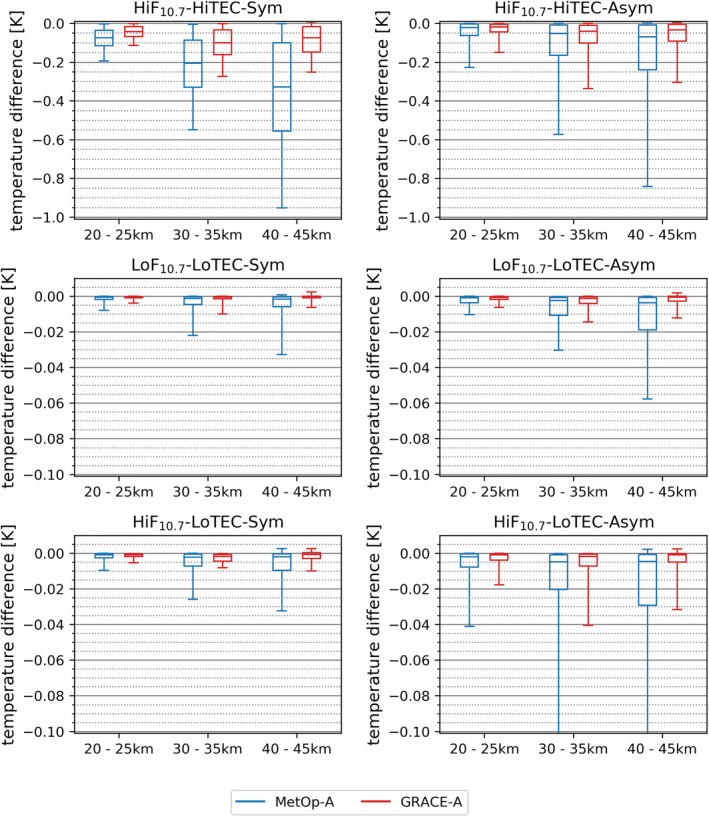
Statistics of the size of the kappa‐correction‐induced temperature deviations versus standard‐correction, for all six characteristic condition cases, comparing the results for MetOp‐A (blue) and GRACE‐A (red) in representative lower (20–25 km), middle (30–35 km), and upper (40–45 km) stratosphere layers. The box‐whisker bars show the median and the quartiles (box, median as horizontal line within) plus the 5th and 95th percentiles (whiskers). The *y*‐axis range is zoomed into by a factor of 10 in the bottom two rows, to enable a legible illustration of the values for these low activity/ionization cases.

To visually summarize the influence of the kappa‐correction term plus the subsequent retrieval process on RO temperature profiles, we show in Figure 7 a statistical results overview of all six characteristic condition cases for both MetOp‐A and GRACE‐A, in the form of box‐and‐whisker plots showing the median/quartiles/5‐95percentile values for lower, middle, and upper stratosphere layers. In line with the discussion before we find that MetOp‐A shows consistently higher deviations than GRACE‐A, most visible in the upper stratosphere (40–45 km layer). For example, for MetOp‐A and the high solar activity near‐symmetry case, the temperature deviation increases from ∼−0.08 K to ∼−0.3 K, for the layers 20–25 km to 40–45 km, respectively. GRACE‐A, on the other hand, stays below ∼−0.1 K. For the low‐level condition cases (low solar activity and low ionization such as during night time and mostly at high latitudes), the temperature deviations stay generally smaller than ∼−0.02 K up to at least the 75th percentile.

Overall this finding clearly signals the fact that the kappa‐correction‐induced temperature profile deviations in the stratosphere significantly depend on both the magnitude of the kappa‐correction term itself as applied to the observed bending angle profile and on the observation‐to‐background weighting or other averaging/smoothing of bending angles, generally performed as a statistical initialization step (Danzer et al., 2020; Gleisner & Healy, 2013; Li et al., 2015; Schwarz et al., 2017) toward the subsequent refractivity and dry‐air retrieval process.

Table 3 finally provides a summary of the deviation results found in this study for the kappa‐correction‐induced deviations at bending angle, refractivity, pressure, and temperature level across the stratosphere, focusing here on the high solar activity and ionization and near‐symmetric case (HiF_10.7_‐HiTEC‐Sym) as well as the high solar activity and low ionization strong‐asymmetry case (HiF_10.7_‐LoTEC‐Asym). The comparison of these two cases illustrates the dependence of the second‐order RIEs on the diurnal cycle. The first case relates to mainly daytime events, with significantly enlarged RIEs, while the second one mainly relates to nighttime events, where the RIEs are rather small. The results for the other cases are given in Appendix A. We consider these beyond a concise numerical summary useful reference numbers also for further studies on the impact of RIEs and their correction in future, in particular when intercomparing different RIE correction methods and when studying more quantitatively the co‐influence of the algorithmic choices in the subsequent retrieval process. An example of the relevance of this co‐influence is evident from comparing the temperature‐deviation results of this study to the predecessor study by Danzer et al. (2020), who used a so‐called averaging‐profile inversion (API) approach that retrieves climatological RO temperature profiles from averaged bending angle profiles: under high solar activity conditions with similar size of the kappa‐correction term at bending angle level those mean stratospheric temperature deviations appear about twice as high compared to the mean deviations from this study.

**Table 3:** *Size of the Kappa‐Correction Term on Bending Angle (* *α* *Profiles and of the Kappa‐Correction‐Induced Relative Refractivity* *N* *, Relative Pressure* *p* *, and Temperature* *T* *Deviations (After WEGC's rOPS Processing as Used in this Study), for Lower, Middle, and Upper Stratospheric Layers and for Both MetOp‐A and GRACE‐A (2008–2015 Data)*

HiF_10.7_ *‐HiTEC‐Sym*								
**MetOp‐A**	Median				Standard deviation			
	*α* [μrad]	*N* [10^−3^%]	*p* [10^−2^%]	*T* [K]	*α* [μrad]	*N* [10^−3^%]	*p* [10^−2^%]	*T* [K]
30–35 km	−0.034	−0.421	−0.131	−0.206	0.030	0.354	0.109	0.174
35–40 km	−0.036	−0.843	−0.205	−0.292	0.030	0.702	0.172	0.254
40–45 km	−0.038	−1.591	−0.292	−0.330	0.031	1.315	0.247	0.309
**GRACE‐A**	*α* [μrad]	*N* [10^−3^%]	*p* [10^−2^%]	*T* [K]	*α* [μrad]	*N* [10^−3^%]	*p* [10^−2^%]	*T* [K]
30–35 km	−0.026	−0.295	−0.073	−0.101	0.035	0.325	0.072	0.094
35–40 km	−0.029	−0.569	−0.111	−0.113	0.034	0.603	0.103	0.111
40–45 km	−0.031	−0.993	−0.093	−0.077	0.033	1.007	0.129	0.093

HiF_10.7_ *‐LoTEC‐Asym*								
**MetOp‐A**	Median				standard deviation			
	*α* [μrad]	*N* [10^−3^%]	*p* [10^−2^%]	*T* [K]	*α* [μrad]	*N* [10^−3^%]	*p* [10^−2^%]	*T* [K]
30–35 km	−0.001	−0.014	−0.004	−0.005	0.030	0.311	0.079	0.113
35–40 km	−0.001	−0.028	−0.006	−0.006	0.030	0.594	0.119	0.149
40–45 km	−0.001	−0.050	−0.008	−0.005	0.030	1.054	0.160	0.152
**GRACE‐A**	*α* [μrad]	*N* [10^−3^%]	*p* [10^−2^%]	*T* [K]	*α* [μrad]	*N* [10^−3^%]	*p* [10^−2^%]	*T* [K]
30–35 km	−0.001	−0.008	−0.002	−0.002	0.014	0.115	0.023	0.026
35–40 km	−0.001	−0.013	−0.002	−0.002	0.012	0.205	0.031	0.028
40–45 km	−0.001	−0.023	−0.003	−0.001	0.011	0.328	0.037	0.020

*Note*: The characteristic condition cases of high solar activity/high Ionization (mainly equatorial/daytime)/near‐symmetry (HiF_10.7_‐HiTEC‐Sym) and of High Solar Activity/Low Ionization (Mainly Non‐equatorial/nighttime)/strong‐asymmetry (HiF_10.7_‐LoTEC‐Asym) are Tabulated here (Further Cases in Appendix A).

### 3.2 Intercomparison of Kappa‐Correction Results With Other Datasets

**Figure 8:**
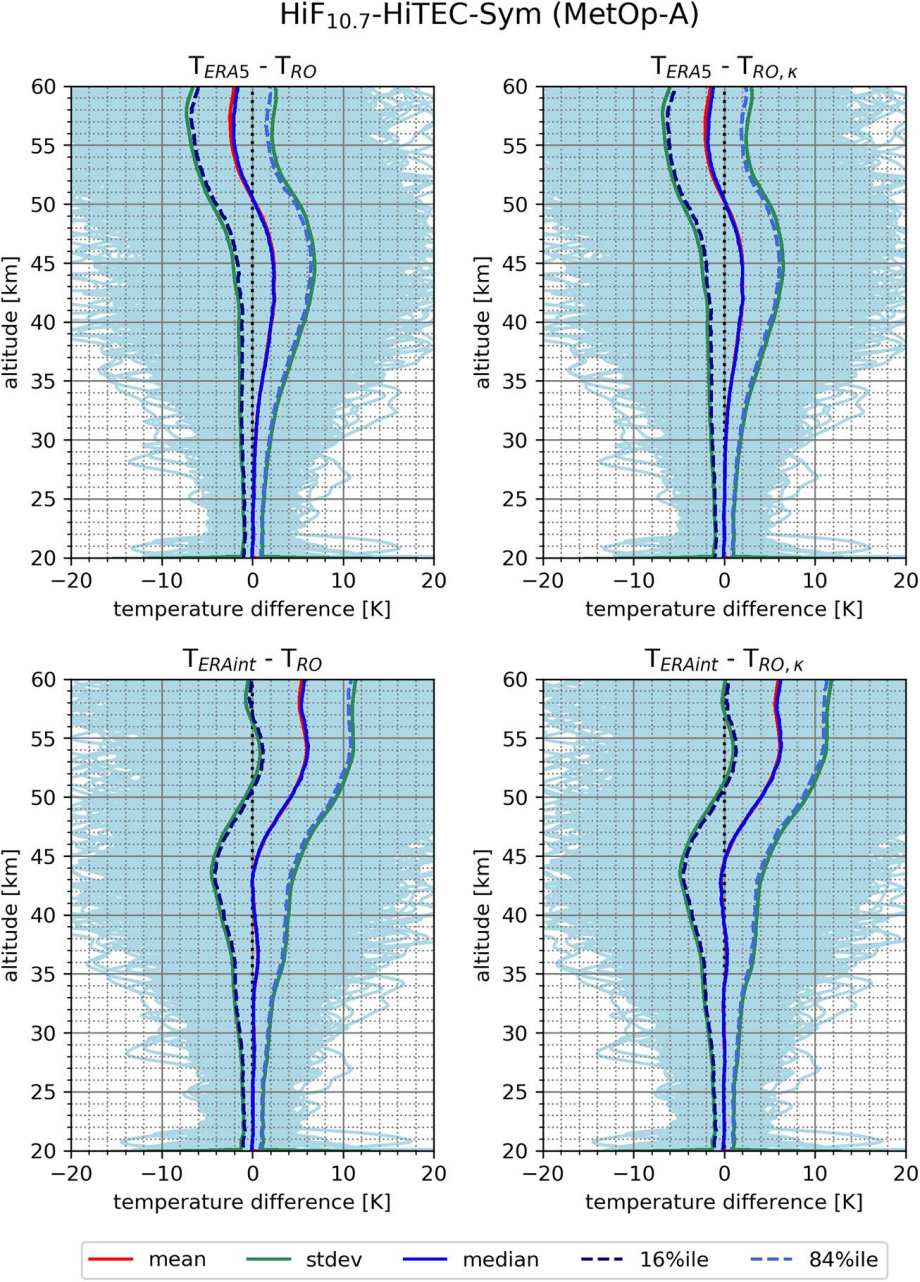
RO temperature profile statistics difference relative to ERA5 (top row) and ERA‐Interim (bottom row) for the condition case HiF_10.7_‐HiTEC‐Sym (MetOp‐A). The difference statistics just with the standard correction (*T*_*RO*_) (left column) and with the kappa‐correction applied (*T*_*RO*,*κ*_) (right column) are shown. The meaning of the depicted statistics, from mean to percentile profiles, can be found in the legend at bottom.

Figure 8 analyzes RO temperature differences relative to comparison datasets from the European reanalyzes ERA5 (top) and ERA‐Interim (bottom), comparing RO‐retrieved stratospheric temperature profile data statistics just with standard‐correction (left) and with the kappa‐correction applied (right), for the case HiF_10.7_‐HiTEC‐Sym. This intercomparison analysis indicates a decrease of the RIE by about ∼0.3 K due to applying the kappa‐correction. This slight mean decrease appears against both the ERA5 and ERA‐Interim datasets.

In general, good agreement between the comparison datasets and RO temperature with both corrections is observed in the lower stratosphere. However, inspecting more closely, applying the kappa‐correction increases the closeness of agreement by about ∼2 km in altitude, against the comparison datasets under this setup. An interesting trait of the temperature difference against ERA5 can be seen at ∼50 km. At this altitude the difference switches its sign and changes from positive to negative. This feature also occurs for the GRACE‐A satellite data (not shown), but at a lower altitude of about 47 km. Another relevant feature of the temperature difference is its general increase with altitude, making it more difficult to attribute the reasons of deviations. In cases of low solar activity, the temperature difference between RO data and the comparison data are found somewhat smaller than in Figure 8 (not shown).

**Figure 9:**
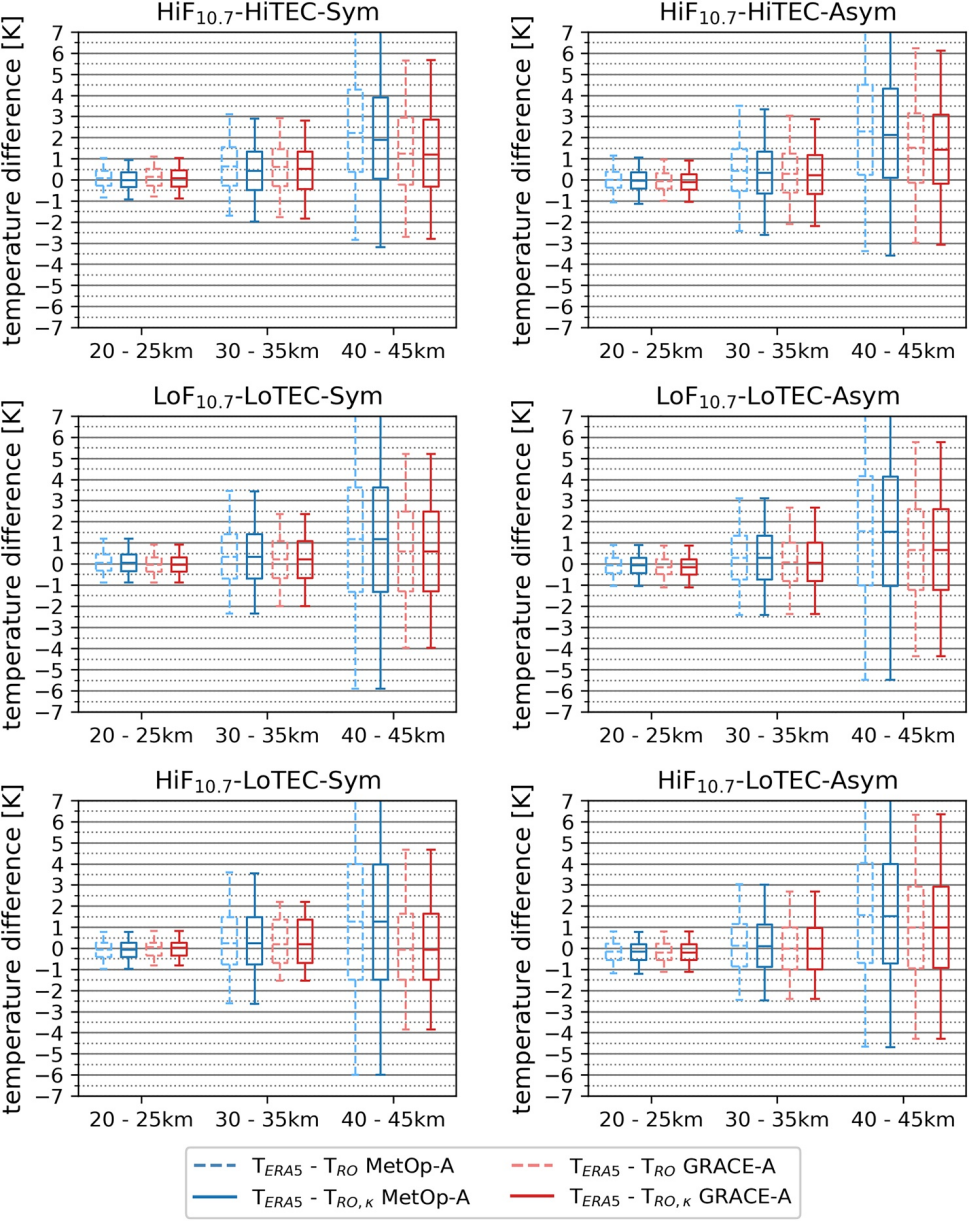
Temperature differences from intercomparison for all six characteristic condition cases, comparing the results for MetOp‐A (blue) and GRACE‐A (red), just with the standard correction (dashed) and with the kappa‐correction applied (solid), with ERA5 as comparison reference dataset. The box‐whisker bars show the median and the quartiles (box, median as horizontal line within) plus the 5th and 95th percentiles (whiskers).

**Figure A1:**
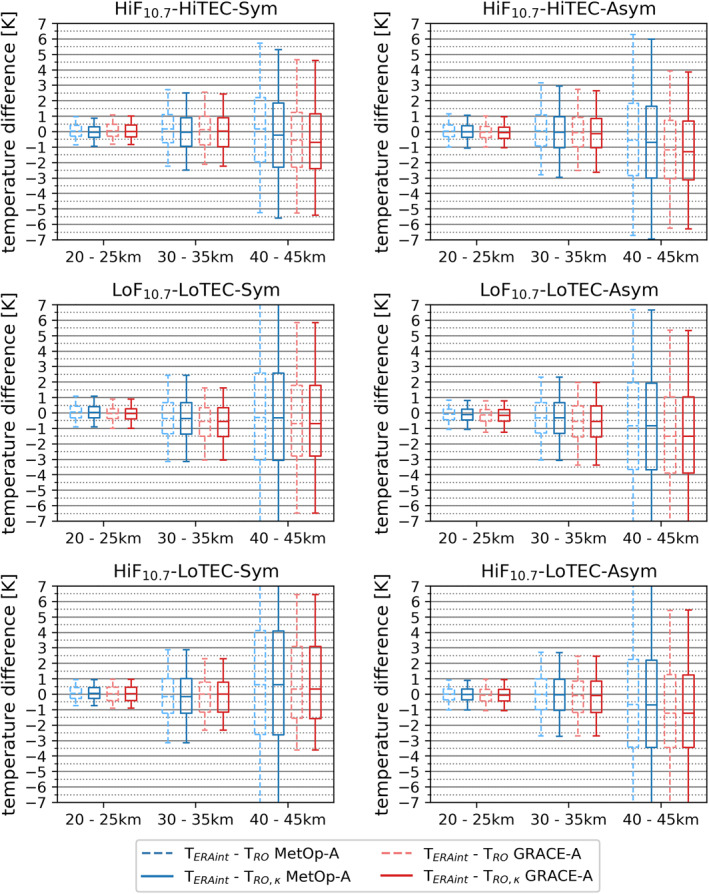
Temperature differences from intercomparison for all six sampling characteristic condition cases, comparing the results for MetOp‐A (blue) and GRACE‐A (red), just with the standard correction (dashed) and with the kappa‐correction applied (solid), with ERA‐Interim as comparison reference dataset. The box‐whisker bars show the median and the quartiles (box, median as horizontal line within) plus the 5th and 95th percentiles (whiskers).

In order to visually summarize the intercomparison information, Figure 9 shows a box‐and‐whisker plot similar to Figure 7, here visualizing the differences of kappa‐corrected (solid lines) and just standard‐corrected (dashed lines) RO temperature data from MetOp‐A (blue) and GRACE‐A (red) across stratospheric layers against the ERA5 data (the same can be found for ERA‐Interim in Appendix A). Besides the previously mentioned traits, the plots additionally reveal that, in the lower and middle stratosphere, the median of the near‐symmetry cases is slightly higher than the one of the asymmetry cases, whereas in the upper stratosphere it is the other way round. The comparison to ERA‐Interim (Figure A1 in Appendix A) exhibits the same characteristics.

The findings of the comparison against both reference datasets can be summarized as follows:

- The temperature difference decreases slightly due to the application of the kappa‐correction by about 0.2–0.4 K, for cases with high solar activity and ionization.
- The temperature difference is in general smaller in the GRACE‐A satellite data than in the MetOp‐A data, but it exhibits the same characteristics.
- In lower stratospheric layers symmetric conditions lead to a higher mean temperature difference than asymmetric conditions do.

Overall we find, in line with the predecessor climatology‐based study (Danzer et al., 2020) that the quality of reference datasets is still marginal for conclusive validation findings for such small effects like the kappa‐correction influence of interest here.

We further inspected co‐located profiles from the Michelson Interferometer for Passive Atmospheric Sounding (MIPAS) middle‐atmosphere dataset, and also the Sounding of the Atmosphere using Broadband Emission Radiometry (SABER) with our RO datasets. However, these datasets were found not suitable for our analysis, as already was shown by Liu et al. (2020). MIPAS faces the practical problem of having no measurements in the most interesting time frame of high solar activity (data exist only to April 2012), while SABER has the feature of a cold bias of 3 K between 20 and 35 km, and near ±2 K between 35 and 45 km altitude (see Innerkofler, 2015) which limits its utility as a reference dataset for the purpose of this study.

## 4 Conclusions

We analyzed the performance of the ionospheric kappa‐correction of radio occultation profiles under diverse ionization and solar activity conditions. Overall, our results are consistent with the results from previous studies; the size of the kappa‐correction strongly depends on the solar activity (Danzer et al., 2013; Liu et al., 2018). We found that:

- The mean of the kappa‐correction term is about 0.05 μrad (high conditions) and smaller than 0.005 μrad (low conditions), which is in line with previous studies based on simulated data (Liu et al., 2015), and small observed data samples (Liu et al., 2020).
- For the kappa‐correction induced RO temperature profile the error at ∼45 km increased from low solar activity and low ionization to high solar activity and high ionization of around ∼−0.005 K to ∼−0.33 K for MetOp‐A, (∼−0.001 K to ∼−0.077 K for GRACE‐A), using the rOPS retrieval system.

Our results indicate that the kappa‐correction induced temperature profile deviations in the stratosphere strongly depend on two main factors: (1) the magnitude of the kappa‐correction term itself, as applied to the observed bending angle profile and (2) on the observation‐to‐background weighting or other averaging/smoothing of bending angles, which is in general performed prior to the refractivity and dry‐air retrieval process.

We note that the differences that we observe in the perturbation of the RIE on temperature level for MetOp‐A data, compared to the climatology study from Danzer et al. (2020), is about a factor of two. However, on bending angle level, the RIE is found to be the same. Hence, we conclude that the differences result from systematic differences in the processing and the retrieval system. Over the past years, there have been some ongoing changes in the rOPS system of the WEGC.

In the comparison of MetOp‐A and GRACE‐A satellite data, we observed (1) different magnitudes for the kappa‐correction and (2) a sign change from negative to positive, for both satellites but at different altitudes. This change of the sign from negative to positive was also observed in the study by Vergados and Pagiatakis (2011), who used GPS RO events of CHAMP to show the impact of the second‐order RIE on atmospheric parameters, however at lower altitudes. In that context we suggest to further investigate the impact of the orbit altitude of satellites on the residual ionospheric error. A multi‐satellite analysis could give some additional insight on that specific feature.

The intercomparison analysis of the study showed the promising result that the application of the kappa‐correction predominantly reduces the temperature error. As comparison datasets we used ERA5, and ERA‐Interim. Under solar high conditions a decrease of the temperature error by a magnitude of ∼0.2 K to ∼0.4 K was found. In general the agreement between RO data and the comparison datasets are very good up to the middle stratosphere between about 30  to 40 km, depending on the comparison dataset. In this setup, we found that the kappa‐correction increased agreement on average by a height of ∼2 km, for both satellites (MetOp‐A and GRACE‐A), in the intercomparison analysis.

In general we note that the quality of reference datasets is still marginal for conclusive validation findings, which makes it difficult to validate possible improvements. Especially since higher‐order ionospheric corrections are quite small. Future investigations will have to include an analysis of the impact of Earth's geomagnetic field. As shown by Blagoveshchensky et al. (2018), sudden changes in the geomagnetic field can lead to a change in the TEC value of up to 67% of its quiet state. A possible way, to assess whether the kappa‐correction accounts for such drastic changes in the geomagnetic field would be a comparison study to the bi‐local correction approach, which includes the strength and the direction of the geomagnetic field (Liu et al., 2020).

The results of Blagoveshchensky et al. (2018) also show that the responses to sudden changes in the magnetic field are very different for different parts of the Earth. We therefore expect that differences average out globally in a climatological context. How changes in the magnetic field affect the quality of the kappa‐correction on individual profiles, needs to be further investigated.

We arrive at the overall conclusion that the simple and fast correction of RO profiles on bending angle level via the ionospheric kappa‐correction is a good alternative to more sophisticated approaches. Since additional background information always comes along with additional biases, the kappa‐correction has the advantage of minimizing the number of such error sources. Despite its simplicity, the kappa‐correction is an important method in operational applications and post‐processing climatological analysis. With our findings we are encouraged to get one step closer to the extension of RO profiles to higher altitudes.

## Data Availability

The OPSv5.6 data are available at the website (https://wegcowncloud.uni-graz.at/s/ZSTT5Ifo3o3iaXn, last access: 18 February 2021). The ERA5 and ERA‐Interim reference data can be downloaded at (https://cds.climate.copernicus.eu/cdsapp#!/dataset/reanalysis-era5-single-levels?tab = overview) and (https://apps.ecmwf.int/datasets/data/interim-full-daily/levtype=ml/). The F_10.7_ solar flux values were downloaded from Natural Resources Canada (https://www.spaceweather.gc.ca/solarflux/sx-en.php, last access: 13 May 2020), the TEC values were extracted from the International Global Positioning System Service (IGS) center (https://kb.igs.org/hc/en-us/articles/115003935351, last access: 23 September 2020).

## Additional Table and Figure

### 1

**Table:** **Table A1**. *Size of the Kappa‐Correction Term on Bending Angle (* *α* *Profiles and of the Kappa‐Correction‐Induced Relative Refractivity* *N* *, Relative Pressure* *p* *, and Temperature* *T* *Deviations (After WEGC's rOPS Processing as Used in this Study), for Lower, Middle, and Upper Stratospheric Layers and for Both MetOp‐A and GRACE‐A (2008–2015 Data)*

*Hi* *F*_10.7_ *‐HiTEC‐Asym*								
**MetOp‐A**	Median				Standard deviation			
	*α* [μrad]	*N* [10^−3^%]	*p* [10^−2^%]	*T* [K]	*α* [μrad]	*N* [10^−3^%]	*p* [10^−2^%]	*T* [K]
30–35 km	−0.011	−0.131	−0.037	−0.053	0.061	0.636	0.171	0.249
35–40 km	−0.011	−0.255	−0.056	−0.071	0.060	1.234	0.262	0.343
40–45 km	−0.012	−0.464	−0.078	−0.071	0.061	2.241	0.364	0.375
**GRACE‐A**	*α* [μrad]	*N* [10^−3^%]	*p* [10^−2^%]	*T* [K]	*α* [μrad]	*N* [10^−3^%]	*p* [10^−2^%]	*T* [K]
30–35 km	−0.012	−0.127	−0.032	−0.042	0.049	0.484	0.115	0.156
35–40 km	−0.012	−0.254	−0.046	−0.048	0.049	0.921	0.168	0.191
40–45 km	−0.013	−0.443	−0.058	−0.035	0.049	1.605	0.216	0.164

*Hi* *F*_10.7_ *‐LoTEC‐Sym*								
**MetOp‐A**	Median				Standard deviation			
	*α* [μrad]	*N* [10^−3^%]	*p* [10^−2^%]	*T* [K]	*α* [μrad]	*N* [10^−3^%]	*p* [10^−2^%]	*T* [K]
30–35 km	−0.0003	−0.007	−0.002	−0.002	0.003	0.047	0.012	0.017
35–40 km	−0.0003	−0.013	−0.003	−0.003	0.004	0.115	0.012	0.020
40–45 km	−0.0003	−0.022	−0.004	−0.002	0.005	0.206	0.021	0.024
**GRACE‐A** *α* [μrad]	*N* [10^−3^%]	*p* [10^−2^%]	*T* [K]	*α* [μrad]	*N* [10^−3^%]	*p* [10^−2^%]	*T* [K]	
30–35 km	−0.0005	−0.008	−0.002	−0.002	0.004	0.040	0.005	0.004
35–40 km	−0.0005	−0.017	−0.003	−0.001	0.004	0.061	0.005	0.007
40–45 km	−0.0005	−0.024	−0.003	−0.001	0.002	0.057	0.006	0.007

*Lo* *F*_10.7_ *‐LoTEC‐Asym*								
**MetOp‐A**	Median				Standard deviation			
	*α* [μrad]	*N* [10^−3^%]	*p* [10^−2^%]	*T* [K]	*α* [μrad]	*N* [10^−3^%]	*p* [10^−2^%]	*T* [K]
30–35 km	−0.0003	−0.006	−0.002	−0.002	0.003	0.033	0.010	0.016
35–40 km	−0.0003	−0.011	−0.003	−0.003	0.003	0.066	0.016	0.023
40–45 km	−0.0004	−0.021	−0.004	−0.004	0.003	0.125	0.023	0.029
**GRACE‐A**	*α* [μrad]	*N* [10^−3^%]	*p* [10^−2^%]	*T* [K]	*α* [μrad]	*N* [10^−3^%]	*p* [10^−2^%]	*T* [K]
30–35 km	−0.0004	−0.005	−0.001	−0.001	0.003	0.025	0.006	0.008
35–40 km	−0.0004	−0.009	−0.002	−0.001	0.003	0.051	0.009	0.010
40–45 km	−0.0004	−0.016	−0.002	−0.001	0.003	0.089	0.011	0.009

*Lo* *F*_10.7_ *‐LoTEC‐Sym*								
**MetOp‐A**	Median				Standard deviation			
	*α* [μrad]	*N* [10^−3^%]	*p* [10^−2^%]	*T* [K]	*α* [μrad]	*N* [10^−3^%]	*p* [10^−2^%]	*T* [K]
30–35 km	−0.0002	−0.004	−0.001	−0.001	0.004	0.037	0.009	0.014
35–40 km	−0.0002	−0.007	−0.002	−0.002	0.004	0.071	0.015	0.021
40–45 km	−0.0002	−0.013	−0.002	−0.002	0.003	0.133	0.021	0.024
**GRACE‐A**	*α* [μrad]	*N* [10^−3^%]	*p* [10^−2^%]	*T* [K]	*α* [μrad]	*N* [10^−3^%]	*p* [10^−2^%]	*T* [K]
30–35 km	−0.0002	−0.003	−0.001	−0.001	0.002	0.016	0.003	0.004
35–40 km	−0.0002	−0.005	−0.001	−0.001	0.002	0.028	0.005	0.006
40–45 km	−0.0002	−0.008	−0.001	−0.000	0.002	0.047	0.006	0.006

*Note*: The characteristic condition cases HiF_10.7_‐HiTEC‐Asym, HiF_10.7_‐LoTEC‐Sym, LoF_10.7_‐LoTEC‐Asym, and LoF_10.7_‐LoTEC‐Sym are Tabulated here\enleadertwodots.
